# Importance of Hospital Performance Indicators in Contracting and Financing Medical Services in Surgical Wards

**DOI:** 10.7759/cureus.66976

**Published:** 2024-08-16

**Authors:** Adriana Vladu, Dana Badau, Lucia Georgeta Daina, Horațiu Paul Domnariu, Cristian Marius Daina

**Affiliations:** 1 Doctoral School, Faculty of Medicine and Pharmacy, University of Oradea, Oradea, ROU; 2 Health Policy Department, Transilvania University of Brasov, Brasov, ROU; 3 Psycho-Neurosciences and Recovery Department, Faculty of Medicine and Pharmacy, University of Oradea, Oradea, ROU; 4 Surgical Disciplines Department, Faculty of Medicine and Pharmacy, University of Oradea, Oradea, ROU

**Keywords:** number of cases, case complexity index, surgical wards, hospital performance, healthcare system, medical services, financial management

## Abstract

In the Romanian healthcare system, public hospitals' contract for the provision of healthcare services with the National Health Insurance Services (NHIS) is the main source of revenue in a hospital's income and expenditure budget. In Romania, for acute diseases, payment is made on a per-case basis for hospitals financed under the diagnostic-related groups (DRG) system, which is calculated according to the indicators achieved. The main objective of the study aimed at the quantitative and comparative analysis of hospital performance indicators used in the calculation of the contracted amount with NHIS, in order to quantify the results and evaluate the effectiveness of the measures taken in the surgical wards of the Emergency Clinical County Hospital of Oradea (ECCHO) compared to the annual average values at the national level, in the period 2012-2022. The indicators, such as the number of beds, number of cases, average length of hospitalization (LOH), and case complexity index (CCI), were analyzed at the level of the ECCHO, a tertiary care hospital in the surgical wards. Rehabilitation and modernization of wards, laboratories, operating theatres, and high-performance equipment have been made possible through efficient management and monthly monitoring of medical and financial activity. The average LOH actually carried out on the surgical wards has been decreasing over the 11 years analyzed, with a lower number of hospital days than at the national level. The CCI achieved by the hospital's surgical wards has had an increasing evolution throughout the period analyzed, higher than the national value. The maximum amount contracted and the amount contracted by the hospital is higher when the LOH and CCI indicators achieved by each section are included in the formula than the indicators established at the national level. A lower LOH and a higher CCI than the national values facilitated the contracting of a higher amount. Optimization of the indicators by hospital performance is correlated with improved funding by the NHIS. Increasing the contracted amount facilitates the contracting of an exponential annual number of cases, resulting in more efficient medical services in the surgical wards.

## Introduction

Reviewing hospital activity is an essential process to improve the efficiency, quality, and safety of patient care. Measuring efficiency enables hospitals to quickly identify problems, optimize processes, and ensure the best outcomes for patients [1]. Data obtained from activity analysis can be used to make informed decisions about hospital management, resource allocation, and implementation of new strategies [2,3]. The significant increase in the cost of hospital services in recent years has led to increased attention from authorities and hospital managers [4], and the financial sustainability of hospitals represents an important component of the security of the provision of medical care [5]. Hospitals provide preventive, curative, and rehabilitative healthcare services to a limited population, but consume around 50-80% of total healthcare expenditure [6]. This has led to the implementation of measures to allocate resources efficiently. Public hospitals in Romania operate on the principle of financial autonomy, organizing their activities on the basis of their own income and expenditure budget. The main source of revenue within the income and expenditure budget is the contract for the provision of medical services of the public hospital with the health insurance fund (the National Health Insurance Services (NHIS)) [7]. The hospital signs a contract for the provision of hospital medical services, on a continuous hospitalization basis, with the health insurance fund, under the terms of the framework contract [8] and the rules of the framework contract [9]. For acute conditions, payment is made based on the rate per solved case (diagnostic-related group (DRG) system), and for chronic diseases, payment is made based on the rate per day of hospitalization. The annual rules of the framework contract provide for a number of hospital-specific indicators: quantitative and qualitative indicators. Quantitative indicators include the following: number of beds, national average bed utilization rate, length of hospital stay, case complexity index, charge per case (DRG), etc. Qualitative indicators include the degree of complexity of hospital medical services provided according to hospitalized morbidity, hospital equipment, and specialist staffing; nosocomial infections in relation to the total number of discharges; degree of operability recorded in the surgical wards/departments; mortality in relation to the total number of discharges; the number of medical/surgical emergencies presented in the emergency structures [9].

Health systems performance is a concept that encompasses the quality of health services and describes the extent to which health systems are able to achieve their objectives [10]. Measuring quality enables improvement actions to be implemented in order to provide better care to patients [11]. The use of standardized indicators leads to a better evaluation of the health services provided, as well as increasing transparency and patient confidence [12]. Performance monitoring has been a concern of the WHO for more than two decades and in 2003 it launched a project to support hospitals in developing a performance measurement framework [12,13].

The continuous improvement of the indicators analyzed in the hospital is the consequence of the careful monitoring of these indicators, in the context of the accreditation of the hospital by the National Authority for Quality Management in Health [14], but also in the desire to provide efficient and quality medical services. Continuous quality improvement is dependent on the hospital's financial resources. Many hospitals in Romania face financial problems caused by both the health system and the specific management of the hospital [15]. The main source of income for a public hospital is the contract with the health insurance company. The total amount contracted by the hospital with the NHIS varies according to a number of factors such as type of hospital (county, municipal, city, emergency, clinical, etc.), type of services provided (acute or chronic hospital care), hospital structure (specialties), and level of competence of the hospital [7]. Within this amount, the hospital must provide health care to a number of cases.

The measures taken by the hospital's management concerned the distribution of the number of cases by departments, informing the heads of departments about the number of cases assigned to them, as well as the contracted amount related to each department. Periodically, the hospital management analyzes the medical and financial activity in each ward. Because indicators made by the hospital are included in the formula of the amount contracted with the health insurance company, additional attention is paid to the number of cases made, the average duration of hospitalization (DMS), and the index of complexity of cases (ICM) [7-9]. The ICM and DMS values achieved by the hospital are compared with the national average values. At the national level, on a platform (drg.ro), the average national values are posted, obtained from the processing of data reported by hospitals in the Single Integrated Information System. These average national values (e.g., DMS and ICM) are indicative, and they do not enter into the formula for calculating the amount contracted by the hospital. ICM and DMS are part of hospital performance indicators, and awareness of their importance is essential from a financial point of view.

Our study was carried out in a county-level emergency hospital in Romania, with a complex structure of medical and surgical specialties [14]. Considering the complex structure of the hospital, only the surgical wards were selected in the study due to the following considerations: the first beneficiaries of important investments in the hospital must support the activity of the operating blocks - rehabilitated and modernized (personnel expenses, medical equipment, current expenses, etc.) and the fact that part of these investments were made from the hospital's own financial funds, funds from the contract with the Health Insurance Company.

The main objective of the study aimed at the quantitative and comparative analysis of hospital performance indicators used in the calculation of the contracted amount with NHIS, in order to quantify the results and evaluate the effectiveness of the measures taken in the surgical wards of the Emergency Clinical County Hospital of Oradea (ECCHO) compared to the annual average values at the national level, in the period 2012-2022.

## Materials and methods

Study design

The study is part of a complex doctoral research aimed at improving the efficiency of medical services in the operating theatre. The present study was carried out in the Emergency Clinical Country Hospital of Oradea, a tertiary care, multi-station hospital (three stationary-building bodies) located in the northwest of Romania. The study was carried out in 2024, by collecting, processing, and analyzing data from the hospital's IT system, InfoWorld, for the period 2012-2022. The analyzed period was selected for the following reasons: relatively stable management at the level of the hospital and wards (which determines a reliable long-term strategy), minimal structural and organizational changes, and important investments in surgical wards and operating blocks (renovations, modernization, and equipment). The research was limited to the analysis of the activity in the surgical wards of Stationary I - a separate building of the hospital, where it exists, as well as surgical and medical wards, ICU wards, and Emergency Reception Unit. Compared to medical wards, surgical wards must also support the activity in the operating room (human resources, equipment, current expenses, maintenance, etc.) through the revenues achieved, which has led us to pay more attention to these wards, in the context of investments what took place in the hospital (rehabilitation and modernization of spaces, high-performance equipment). The data provided by the services, such as statistics, informatics, and financial accounting, were collected and processed in the period 2012-2022. During the period analyzed, the total number of beds per hospital ranged from a minimum of 861 beds (2022) to a maximum of 1007 beds (2012), of which, in Ward I, the number of beds decreased from 300 beds (2012) to 258 beds (2020). The surgical wards/compartments analyzed were as follows: general surgery (two wards), cardiovascular surgery, thoracic surgery, vascular surgery, maxillofacial surgery, otorhinolaryngology, plastic surgery, burns, neurosurgery, ophthalmology, orthopaedics and traumatology (two wards), and urology.

The study was approved by the Ethics Committee no. 25323 of 12.10.2018 of the ECCHO, IRB 25323.1/12.10.2018 of the ECCHO. The study complied with all the policies and recommendations of the Declaration of Helsinki of 2008 with its subsequent amendments.

Efficiency indicators analyzed in the study

All wards reviewed provide acute services funded under the DRG system. The DRG is a diagnostic group classification system that includes the cost of resources consumed for patient care, linking patient types to hospital expenditure [16]. In order to achieve the objective of the study research, hospital performance indicators [17] were included in the study and used to calculate the number of acute hospital services paid on a per-case basis (DRG) for DRG-funded hospitals.

The following indicators were analyzed: the number of contractable beds (Nr_pat); the average length of hospitalization actually achieved for reported and validated cases (LOH); and CCI (case-mix index/case complexity index) at the hospital level achieved in the previous year. The following formula was used to calculate the possible contracted amount (CA) per ward/specialty according to the indicators achieved:

SC = P x Case_number x CCI x WCC [8].

The value of the reference percentage (P) is established in relation to the classification of hospitals according to competence [16]. For the hospital analyzed, category II, P = 82%.

Case_number represents the number of cases to be contracted, from the formula:

Case_number = Bed_number x National BUI / LOH.

In the formula, BUI represents the bed use index (man-days hospitalization of inpatients and outpatients / average number of beds) and has, for the acute wards/departments, the value of 290 days, which leads to a utilization rate = 79.45%.

The weighted case charge (WCC) is the reimbursement amount per weighted case at the hospital level and is set each year by the implementing rules of the framework contract. The WCC value set by the rules for Emergency Clinical Country Hospital of Oradea was 1,600 ron (approx. 330 euro) in the period 2012-2020 and 1,854 ron (approx. 380 euro) in the period 2021-2022.

National average values for LOHs and CCI were collected from the DRG platform indicators [17]. At the national level, the average national values by type of hospital and by type of department. The comparative analysis carried out took into account the data published at the national level on the type of ward in a county emergency clinical hospital that provides acute medical services. To compare the hospital LOH values with the national LOH values, the actual LOH per discharged patient was used, which includes all days of hospitalization for a discharged patient.

Statistical analysis

The data collected were registered in Excel (Microsoft® Corp., Redmond, WA) and Medcalc software (https://www.medcalc.org/), which were also used for statistical analysis. The value p<0.05 was considered statistically significant. The results were processed with Statistical Product and Service Solutions (SPSS, version 24; IBM SPSS Statistics for Windows, Armonk, NY) using the following statistical parameters: chi-square (Chi2) and degrees of freedom (df). The existence of a statistically significant relationship between variables in relation to the study hypothesis was demonstrated using chi-square.

## Results

Surgical wards have an important share in the first ward of the ECCHO, representing between 49.72% and 55.05% of the total number of beds, with 12 specialties structured in 14 wards and compartments (Table 1). Pre- and postsurgical LOH was calculated (Table 2).

**Table 1:** Evolution of the number of surgical beds at the Emergency Clinical County Hospital of Oradea (Emergency Clinical County Hospital of Oradea) (2012-2022)

Section/Department	Year										
	2012	2013	2014	2015	2016	2017	2018	2019	2020	2021	2022
	N (%)	N (%)	N (%)	N (%)	N (%)	N (%)	N (%)	N (%)	N (%)	N (%)	N (%)
General surgery	94 (31.33)	94 (31.33)	91 (30.43)	89 (29.86)	91 (30.33)	93 (30.79)	69 (25.55)	65 (24.43)	64 (24.8)	65 (24.43)	65 (24.25)
Cardiovascular surgery	0 (0)	0 (0)	0 (0)	0 (0)	0 (0)	0 (0)	0 (0)	2 (0.75)	4 (0.15)	4 (0.15)	4 (0.14)
Thoracic surgery	6 (2)	6 (2)	6 (2)	6(2.01)	6 (2)	6 (1.98)	6 (2.22)	6 (2.25)	6 (2.32)	6 (2.25)	6 (2.23)
Vascular surgery	0 (0)	0 (0)	3 (1)	5 (1.67)	5 (1.66)	5 (1.65)	5 (1.85)	5 (1.87)	5 (1.93)	5 (1.87)	5 (1.86)
Maxillofacial surgery	15 (5)	15 (5)	15 (5.01)	15 (5.03)	15 (5)	15 (4.96)	15 (5.55)	15 (5.63)	14 (5.42	14 (5.63)	15 (5.59)
Otorhinolaryngology	15 (5)	15 (5)	15 (5.01)	15 (5.03)	15 (5)	15 (4.96)	15 (5.55)	15 (5.63)	15 (5.81)	14 (5.63)	15 (5.59)
Plastic surgery	20 (6.66)	20 (6.66)	20 (6.68)	20 (6.71)	20 (6.66)	20 (6.62)	20 (7.4)	20 (7.51)	20 (7.75)	20 (7.51)	20 (7.46)
Burn unit	5 (1.66)	5 (1.66)	5 (1.67)	5 (1.67)	5 (1.66)	5 (1.65)	5 (1.85)	5 (1.87)	5 (1.93)	5 (1.87)	5 (1.86)
Neurosurgery	25 (8.33)	25 (8.33)	25 (8.36)	25 (8.38)	25 (8.33)	25 (8.27)	25 (9.25)	25 (9.39)	25 (9.68)	25 (9.39)	25 (9.32)
Ophthalmology	10 (3.33)	10 (3.33)	10 (29.9)	10 (3.35)	10 (3.33)	10 (3.31)	10 (3.7)	10 (3.75)	10 (3.87)	10 (3.75)	10 (3.73)
Orthopaedics traumatology	65 (21.66)	65 (21.66)	64 (21.4)	63 (21.47)	63 (21)	63 (20.86)	63 (23.33)	63 (23.68)	55 (21.31)	63 (23.68)	63 (23.5)
Urology – N(%)	45 (15)	45 (15)	45 (15.05)	45 (15.1)	45 (15)	45 (14.9)	37 (13.7)	35 (13.15)	35 (13.56)	35 (13.15)	35 (13.05)
Total surgical specialty beds, N(%)	300	300	299	298	300	302	270	266	258	266	268
% surgical specialty beds of total beds	55.05	53.86	53.78	53.69	53.83	54.00	51.23	50.86	50.10	49.72	50.76

**Table 2:** Evolution of hospital (ECCHO) LOH and national LOH between 2012 and 2022 ECCHO, Emergency Clinical County Hospital of Oradea

Section/Compartment	Year										
	2012	2013	2014	2015	2016	2017	2018	2019	2020	2021	2022
Achieved LOH – hospital (ECCHO)											
General surgery	5.37	4.92	4.71	4.84	4.51	4.77	4.77	4.91	6.15	6.18	5.54
Cardiovascular surgery	0	0	0	0	0	0	0	7.5	11.15	7.36	7.92
Thoracic surgery	7.26	7.15	6.34	9.78	6.77	8.61	7.94	7.8	6.78	6.33	5.87
Vascular surgery	0	0	4.11	5.79	5.5	5.25	5.47	5.1	8.63	6.37	6.36
Maxillofacial surgery	6.14	5.36	4.47	3.44	3.46	3.67	3.44	2.97	3.86	4.78	3.35
Otorhinolaryngology	4.7	4.51	4.82	4.36	4.48	4.3	4.05	4.04	4.76	4.33	4.2
Plastic surgery	5.92	4.54	3.6	4.55	4.25	4.61	4.48	4.13	4.36	4.35	3.93
Burn unit	9.11	8	6.25	9.58	6.79	9.55	6.83	8.7	10.79	14.81	11.6
Neurosurgery	7.25	6.99	7.36	6.23	6.21	6.11	5.91	6.17	6.9	6.37	7.08
Ophthalmology	3.76	2.94	2.42	1.5	1.51	1.5	1.47	1.28	1.58	1.95	1.38
Orthopaedics traumatology	6.43	5.8	5.38	5.97	4.76	4.68	5.18	4.93	4.41	4.15	4.53
Urology	5.21	5.21	4.61	4.9	4.23	7.88	4.31	3.76	3.48	3.53	3.47
Total surgical wards	5.70	5.22	4.86	5.01	4.75	5.19	4.66	4.48	5.10	4.95	4.67
National LOH											
General surgery	6.68	6.78	6.82	6.79	6.73	6.55	6.47	6.36	6.81	6.52	6.15
Cardiovascular surgery	0	0	0	0	0	0	0	7.99	8.68	8.69	9.21
Thoracic surgery	7.9	8.99	8.56	8.36	8.54	8.14	8.03	7.64	7.73	7.36	7.26
Vascular surgery	0	0	7.26	7.11	7.19	6.89	6.81	6.7	7.21	7.14	6.3
Maxillofacial surgery	4.47	4.58	4.34	4.25	4.24	4.24	4.17	4.13	4.22	4.17	3.98
Otorhinolaryngology	5.11	5.49	5.2	5.15	5.17	5.02	4.88	4.77	4.97	4.62	4.11
Plastic surgery	5.52	5.51	5.89	5.95	5.83	5.44	5.29	5.17	5.73	5.11	4.83
Burn Unit	12.25	12.27	11.4	11.09	12.34	13.86	12.47	12.48	15.36	16.04	13.07
Neurosurgery	7.59	6.6	6.85	7.04	7.19	7.23	7.25	7.1	7.72	7.33	7.12
Ophthalmology	3.23	3.48	3.31	3.33	3.33	3.06	3.12	3.03	3.03	2.83	2.68
Orthopaedics Traumatology	7.07	7.05	7.01	7.06	7.12	6.86	6.78	6.66	6.69	6.18	6.08
Urology	5.53	6.18	6.05	5.91	5.72	6.45	5.31	5.2	5.12	4.93	4.71
Total surgical wards	6.32	6.43	6.44	6.39	6.38	6.35	6.02	5.87	6.27	5.96	5.57
LOH achieved hospital (ECCHO) compared to national LOH (%)											
General surgery	-19.57	-27.42	-30.87	-28.67	-32.96	-27.18	-26.22	-22.90	-9.64	-5.24	-9.93
Cardiovascular surgery	0	0	0	0	0	0	0	-6.13	28.42	-15.28	-13.99
Thoracic surgery	-8.06	-20.47	-25.97	16.99	-20.79	5.77	-1.12	2.11	-12.27	-14.01	-19.21
Vascular surgery	0	0	-43.36	-18.57	-23.47	-23.80	-19.68	-23.95	19.72	-10.79	0.90
Maxillofacial surgery	37.41	17.03	3.02	-19.06	-18.27	-13.44	-17.51	-28.06	-8.63	14.51	-15.83
Otorhinolaryngology	-7.96	-17.85	-7.36	-15.34	-13.36	-14.34	-17.01	-15.32	-4.13	-6.28	2.30
Plastic surgery	7.18	-17.60	-38.85	-23.53	-27.06	-15.26	-15.31	-20.18	-23.99	-14.98	-18.75
Burn unit	-25.62	-34.80	-45.18	-13.62	-44.98	-31.10	-45.23	-30.22	-29.75	-7.71	-11.28
Neurosurgery	-4.51	5.91	7.50	-11.51	-13.73	-15.49	-18.48	-13.10	-10.66	-13.06	-0.57
Ophthalmology	16.28	-15.52	-26.94	-54.95	-54.50	-50.98	-52.88	-57.81	-47.77	-31.30	-48.54
Orthopaedics traumatology	-9.00	-17.78	-23.23	-15.49	-33.13	-31.84	-23.60	-26.04	-34.03	-32.87	-25.46
Urology	-5.78	-15.70	-23.86	-17.09	-26.05	22.17	-18.83	-27.78	-31.94	-28.34	-26.33
Total surgical wards	-9.80	-18.77	-24.49	-21.59	-25.63	-18.32	-22.71	-23.72	-18.55	-16.85	-16.20

Since 2015, hospitals have also been obliged to report pre- and postoperative LOH to the National Institute for Health Services Management (INMSS) - Centre for Human Resources Evaluation and Analysis of Health Services, where, after collecting, validating, and centralizing these data, national averages are established.

According to Table 1, in the period 2012-2022, the number of surgical beds ranged from 258 in 2020 to 302 in 2017. The average number of surgical beds during the study period was 284, representing 52.50% of the total beds. The share of surgical beds ranged from 49.72% in 2021 to 55.05% in 2012. In 2020 and 2021, a reorganization of wards was required due to the pandemic. General surgery and orthopaedics-traumatology specialties are divided into two wards. Vascular surgery was established in 2014 and cardiovascular surgery in 2019. The share of surgical beds had a decreasing trend in the period 2012-2022, mainly due to the fact that Station I entered into an extensive rehabilitation, modernization, and reorganization process, and the COVID-19 pandemic years required the organization of COVID wards/departments.

The average length of hospitalization actually performed on the surgical wards in the ECCHO, in the period 2012-2022 had a decreasing trend, with some increasing variations in the years 2017 and 2020. The highest LOH was recorded in 2012 in all surgical wards/departments analyzed. Compared to the LOH at the national level, a lower number of hospital days were recorded at the hospital level (Table 2). From the analysis by hospital wards, and by specialty type, the highest LOH were on burns (9.27 days), cardiovascular surgery (8.48 days), thoracic surgery (7.33 days), and neurosurgery (6.59 days) (Table 2). From 2012 to 2022, the average length of hospital stay was 4.96 days. The average length of hospitalization showed a decreasing sinusoidal trend, decreasing significantly from 2014 (from 5.70 days to 4.86 days, 95%CI: 0.195-1.128, χ2= 7.757, p=0.005), reaching 4.67 days in 2022 (95%CI: 0.569-1.529, χ2= 18.139, p<0.001).

Nationally, the LOH followed a nearly flat curve from 2012 to 2017 (6.32 days -6.44 days). Since 2018, the national LOH followed a downward sinusoidal curve (from 6.02 days to 5.57 days, p=0.112). Regardless of the year, the realized LOH was significantly lower than the national LOH (p<0.05).

At the hospital level, by surgical wards, the achieved LOH was below the national LOH value from 2012 to 2022, with the largest difference found in 2016 (approx. -25%) and the smallest in 2012 (approx. -10%). Pre- and post-surgical LOH was calculated (Table 3).

**Table 3:** Pre- and postoperative LOH evolution (2012-2022) - hospital (ECCHO)

Section/Department	Year										
	2012	2013	2014	2015	2016	2017	2018	2019	2020	2021	2022
Presurgical LOH – hospital (ECCHO)											
General surgery	1.16	1.06	1.02	1.09	1.12	1.16	1.21	1.19	1.78	1.59	1.33
Cardiovascular surgery	0	0	0	0	0	0	0	5.00	2.88	1.95	2.76
Thoracic surgery	3.42	2.95	3.60	3.12	5.41	3.46	3.50	2.66	3.49	2.02	1.26
Vascular surgery	0	0	1.39	1.43	1.79	1.23	1.47	1.73	3.56	2.36	2.25
Maxillofacial surgery	1.74	1.37	1.16	1.33	1.12	1.15	1.14	0.92	1.63	1.78	1.12
Otorhinolaryngology	0.95	1.10	1.02	1.07	1.11	1.08	1.01	1.01	1.72	1.46	1.26
Plastic surgery	1.55	1.44	1.01	1.03	0.99	0.84	0.85	0.88	1.23	1.14	0.99
Burn unit	1.90	2.44	2.23	2.18	1.78	1.59	1.56	1.72	2.13	4.22	3.26
Neurosurgery	6.91	7.14	5.48	5.27	3.64	2.91	2.53	2.56	3.04	2.44	2.67
Ophthalmology	1.12	0.99	0.63	0.55	0.29	0.25	0.21	0.17	0.60	0.82	0.12
Orthopaedics traumatology	1.77	1.60	1.41	1.43	1.61	1.45	1.42	1.44	1.74	1.54	1.47
Urology	2.02	2.00	1.71	1.69	1.59	1.53	1.61	1.31	1.39	1.41	1.31
Total surgical wards	1.72	1.53	1.36	1.35	1.40	1.31	1.31	1.24	1.75	1.58	1.37
Postsurgical LOH – hospital (ECCHO)											
General surgery	4.73	4.37	4.17	4.16	4.27	4.25	4.07	4.29	5.32	5.34	4.61
Cardiovascular surgery	0	0	0	0	0	0	0	8.00	11.75	5.68	5.43
Thoracic surgery	4.99	5.13	6.24	4.79	8.23	4.19	4.95	5.29	3.95	4.91	5.34
Vascular surgery	0	0	3.01	4.16	4.35	4.11	3.86	3.66	5.50	4.75	4.58
Maxillofacial surgery	4.89	4.19	3.38	2.85	2.32	2.26	2.26	2.02	2.27	3.08	2.23
Otorhinolaryngology	3.10	3.16	3.33	2.59	2.98	2.88	2.54	2.78	3.17	2.83	2.71
Plastic surgery	4.26	3.09	2.57	2.82	3.21	3.03	3.44	3.04	3.06	3.16	2.96
Burn unit	7.33	5.48	4.14	5.14	5.10	4.90	5.28	6.79	8.43	11.14	8.50
Neurosurgery	9.97	9.85	10.35	10.95	9.80	8.58	8.13	7.58	7.13	7.01	7.67
Ophthalmology	2.24	1.87	1.67	1.35	1.12	1.10	1.16	1.01	0.92	1.00	1.13
Orthopaedics traumatology	4.99	4.73	4.45	4.13	3.90	3.93	3.95	3.75	2.82	2.72	3.19
Urology	3.81	3.86	3.41	3.35	3.13	3.45	3.06	2.63	2.26	2.21	2.21
Total surgical wards	4.65	4.28	4.03	3.86	3.89	3.77	3.62	3.51	3.64	3.64	3.47

Between 2012 and 2019, the duration of preoperative hospitalization decreased statistically significantly from 1.72 days in 2012 to 1.24 days in 2019 (p=0.003), then increased statistically significantly in 2020 (pandemic year) to 1.75 days (p=0.006), and decreased statistically insignificantly in 2021 to 1.58 days (p=0.439). The decreasing trend is also recorded in 2022, 1.37 days (p=0.049).

Postsurgical hospitalization duration decreased statistically significantly from 4.65 days in 2012 to 3.51 days in 2019 (p<0.001), then increased statistically insignificantly in 2020 (pandemic year) to 3.64 days (p=0.658), and decreased statistically insignificantly in 2022 to 3.47 days (p=0.561).

Since 2015, hospitals have also been obliged to report pre- and postoperative LOH to the INMSS - Centre for Human Resources Evaluation and Analysis of Health Services, where, after collecting, validating, and centralizing these data, national averages are established. The data reported by the hospital and those obtained at the national level are represented in Table 4.

**Table 4:** Pre- and postsurgical comparative LOH – hospital (ECCHO) - national (2015-2022) ECCHO, Emergency Clinical County Hospital of Oradea

LOH	Year							
	2015	2016	2017	2018	2019	2020	2021	2022
Presurgical LOH								
Hospital (ECCHO)	1.35	1.4	1.31	1.31	1.24	1.75	1.58	1.37
National	1.74	1.79	1.78	1.73	1.75	1.91	1.82	1.69
p	0.017	0.019	0.004	0.013	0.002	0.502	0.263	0.056
Postsurgical LOH								
Hospital (ECCHO)	3.86	3.89	3.77	3.62	3.51	3.64	3.64	3.47
National	4.67	4.46	4.42	4.19	3.99	4.40	4.00	3.84
p	0.003	0.003	0.013	0.032	0.044	0.030	0.257	0.148

Preoperative hospitalization duration performed by the ECCHO was statistically significantly lower than the national level during 2015-2019 (p<0.05) and statistically insignificantly lower during the 2020-2022 pandemic period (1.75 days vs 1.91 days, p=0.502, respectively 1.58 days vs 1.82 days, p=0.263) (Table 4). Postsurgical hospitalization duration achieved by the ECCHO was statistically significantly lower than the national level during 2012-2020 (p<0.05) and statistically insignificantly lower in 2021 and 2022 (3.64 days vs 4.00 days, p=0.257. The CCI performed by the hospital's surgical wards shows an increasing trend in the period 2012-2022, from 1.3972 in 2012 to 2.9255 in 2022. The same upward trend is recorded at the national level, from 1.5353 in 2012 to 2.4222 in 2022 (Table 5).

**Table 5:** Evolution of the CCI 2012-2022 (achieved hospital (ECCHO)/national) ECCHO, Emergency Clinical County Hospital of Oradea; CCI, case complexity index

Section/Compartment	Year										
	2012	2013	2014	2015	2016	2017	2018	2019	2020	2021	2022
Achieved CCI - hospital (ECCHO)											
General surgery	1.61	1.59	1.56	1.52	1.70	1.75	1.83	1.98	2.06	2.26	2.51
Cardiovascular surgery	0	0	0	0	0	0	0	3.59	4.48	3.59	4.30
Thoracic surgery	2.20	2.31	2.89	2.70	2.93	3.48	3.57	3.89	3.74	3.60	3.39
Vascular surgery	0	0	1.94	2.19	2.74	2.59	2.39	2.92	3.20	3.16	3.14
Maxillofacial surgery	1.67	1.75	1.72	1.30	1.39	1.30	1.17	2.50	2.30	2.33	2.62
Otorhinolaryngology	1.09	1.45	1.30	2.42	1.67	1.42	1.69	2.17	2.32	2.25	3.02
Plastic surgery	1.10	1.54	1.68	1.69	2.07	3.05	3.03	3.52	3.37	3.41	4.01
Burn unit	2.54	2.64	3.34	3.68	4.22	2.43	4.40	5.03	7.12	5.27	6.14
Neurosurgery	1.40	1.39	1.54	1.79	2.12	2.59	2.77	3.38	3.87	3.61	3.80
Ophthalmology	0.62	0.67	0.70	0.66	0.72	0.75	0.79	0.80	0.78	0.77	0.75
Orthopaedics traumatology	1.34	1.52	1.58	1.81	1.92	2.11	2.15	2.17	2.54	2.75	3.11
Urology	1.12	1.17	1.17	1.27	1.33	1.57	1.38	2.34	2.68	2.72	2.88
Total surgical wards	1.39	1.49	1.63	1.65	1.81	1.94	1.97	2.17	2.63	2.68	2.92
National CCI											
General surgery	2.00	1.61	1.66	1.70	1.78	1.90	1.88	1.97	2.193	2.19	2.12
Cardiovascular surgery	0	0	0	0	0	0	0	3.55	4.31	4.48	4.52
Thoracic surgery	2.214	2.06	2.06	2.06	2.19	2.30	2.24	2.23	2.39	2.27	2.37
Vascular surgery	0	0	2.51	2.53	2.63	2.70	2.69	2.65	2.87	3.03	2.66
Maxillofacial surgery	1.40	0.97	1.25	1.13	0.99	1.10	1.02	1.27	1.57	1.53	1.48
Otorhinolaryngology	1.15	1.49	1.49	1.53	1.55	1.45	1.46	1.47	1.79	1.77	1.52
Plastic surgery	1.05	1.58	1.66	1.66	1.731	1.84	1.98	2.05	2.07	2.20	2.23
Burn unit	2.81	4.18	4.00	3.77	3.89	2.77	3.41	3.91	5.27	4.84	4.02
Neurosurgery	1.72	2.25	2.36	2.44	2.55	2.72	2.68	2.69	3.09	3.14	2.88
Ophthalmology	0.74	0.71	0.72	0.71	0.72	0.72	0.73	0.74	0.75	0.75	0.74
Orthopaedics traumatology	1.16	1.49	1.57	1.60	1.76	1.96	1.84	1.80	1.98	2.07	2.10
Urology	1.13	1.13	1.16	1.19	1.21	1.67	1.32	1.44	1.61	1.71	1.64
Total surgical wards	1.53	1.54	1.62	1.64	1.72	1.86	1.78	1.89	2.28	2.26	2.42
Achieved hospital (ECCHO) CI compared to national CCI (%)											
General surgery	-19.70	-0.86	-6.18	-10.57	-4.66	-7.58	-2.51	0.36	-5.94	3.33	18.27
Cardiovascular surgery	0	0	0	0	0	0	0	1.08	3.96	-19.84	-4.73
Thoracic surgery	-0.31	11.85	40.24	31.15	33.69	51.23	59.51	74.32	56.78	58.24	43.10
Vascular surgery	0	0	-22.57	-13.33	4.13	-4.30	-11.11	9.98	11.61	4.30	17.99
Maxillofacial surgery	19.61	80.09	37,02	14.86	40.91	18.14	15.23	96.93	45.94	52.34	76.55
Otorhinolaryngology	-4.97	-2.59	-13.01	58.29	7.75	-2.12	15.92	47.18	29.88	27.58	98.03
Plastic surgery	4.87	-2.85	1.13	1.72	19.57	66.00	52.42	72.04	62.14	54.98	79.80
Burn unit	-9.71	-36.80	-16.29	-2.43	8.41	-12.10	29.05	28.52	35.02	8.88	52.79
Neurosurgery	-18.31	-38.06	-34.65	-26.72	-17.09	-4.74	3.33	25.89	25.06	15.00	31.74
Ophthalmology	-15.64	-5.45	-2.52	-6.80	0.39	4.98	7.37	7.31	4.80	2.73	1.51
Orthopaedics traumatology	15.82	1.79	0.21	13.39	9.26	7.70	17.10	20.26	28.37	33.15	48.29
Urology	-0.88	3.38	0.60	6.78	10.08	-5.74	4.79	62.43	66.73	58.38	74.97
Total surgical wards	-8.99	-3.48	0.17	0.90	5.17	4.29	11.05	14.83	15.05	18.61	20.78

In terms of the realized CCI, it highlights that, in the first two years of the study period (2012-2013), the CCI value was below the national average (1.3972 vs 1.5353, respectively, 1.4936 vs 1.5475). Since 2014 the realized CCI has been above the national one, from 1.6307 vs 1.6280 to 2.9255 vs 2.4222. The coding of the complexity of cases treated in hospitals has been continuously improved both by a greater focus on principal and secondary diagnoses and by identifying and quantifying cases treated in the ICU (ventilated patients). Compared to -8.99% in 2012 of the national value, the CCI increased at the end of the period under review by 20.78% above the national CCI.

Case complexity differs by surgical section according to specialty. In the last four years analyzed (2019-2022), the CCI is higher in all surgical sections of the hospital compared to national values, except for the cardiovascular surgery section. From the analysis by hospital wards, and by type of specialty, the highest CCI was on the artery (4.2594), cardiovascular surgery (3.9975), thoracic surgery (3.1604), and vascular surgery (2.7014) (Table 5). It should be noted that the first three listed wards also have the highest average length of hospitalization.

**Table 6:** Number of cases and amount contracted (hospital - ECCHO) for 2021 according to indicators achieved per section BUI, bed use index; CCI, case complexity index; ECCHO, Emergency Clinical County Hospital of Oradea; LOH, length of hospitalization

Section	N (%) of beds 2021	National BUI	LOH 2020	Case no.	CCI 2020	Maximum amount, 2021 lei (*euro)	Contracted amount, 2021 lei (*euro)	No. of cases contracted, 2021
A	B	C	D	E	F	G	H	I
Formula				[B]*[C]/[D]		[E]*[F]*WCC	[G]*P	[H]/[F]/WCC
General surgery	65 (24.43)	290.0	6.15	3065	2.0639	11728287.94 *2383604.57	9617196.11 *1954555.74	2513
Cardiovascular surgery	4 (1.5)	290.0	11.15	104	4.4879	865637.42 *175928.26	709822.69 *144261.17	85
Thoracic surgery	6 (2.25)	290.0	6.78	257	3.7496	1784079.59 *362588.32	1462945.26 *297322.42	210
Vascular surgery	5 (1.87)	290.0	8.63	168	3.2096	999810.86 *203197.06	819844.90 *166621.59	138
Maxillofacial surgery	14 (5.26)	290.0	3.86	1052	2.3041	4493138.26 *913165.24	3684373.37 *748795.49	862
Otorhinolaryngology	14 (5.26)	290.0	4.76	853	2.3293	3683445.41 *748606.90	3020425.23 *613857.65	699
Plastic surgery	20 (7.51)	290.0	4.36	1330	3.3724	8317452.22 *1690401.63	6820310.82 *1386129.34	1091
Burn unit	5 (1.87)	290.0	10.79	134	7.1219	1774402.57 *360621.61	1455010.11 *295709.72	110
Neurosurgery	25 (9.39)	290.0	6.90	1051	3.8720	7542824.35 *1532969.74	6185115.97 *1257035.19	862
Ophthalmology	10 (3.75)	290.0	1.58	1835	0.7873	2679112.14 *544490.72	2196871.95 *446482.38	1505
Orthopaedics traumatology	63 (23.68)	290.0	4.41	4143	2.5419	19523970.77 *3967964.14	16009656.03 *3253730.59	3397
Urology	35 (13.15)	290.0	3.48	2917	2.6881	14535900.75 *2954211.19	11919438.62 *2422453.17	2392
Total surgical wards	266 (100)	290.0	5.10	16909	2.6331	77928062.27 *15837749.42	63901011.06 *12986954.52	13865

Taking into account the values achieved by the hospital and the national values of the two indicators: LOH and CCI. The influence of these indicators on the amounts contracted (AC) with the health insurance company was analyzed. Considering the formula for contracting the amount of hospital services, presented in the material and method section, and the fact that the value of the two indicators can influence the AC, it was calculated to what extent the indicators achieved influence the number of cases and the AC. It should be noted that the hospital contracts amounts based on the average of these values and not separately per ward. On the other hand, the performance indicators achieved in excess of the national values ensure that the hospital receives a higher amount when re-contracting. The contracted amount is higher if the number of contracted beds increases, the LOH decreases, and the CCI value increases. Tables 6-7 present the calculated number of cases and the contracted amount for the year 2021, with the value WCC=1.854 lei; in Table 6, it has been calculated per ward according to the achieved indicators, and in Table 7, it has been calculated according to the achieved indicators established in the rules.

**Table 7:** Number of cases and amount contracted (hospital – ECCHO) for 2021 according to the indicators set by the rules BUI, bed use index; CCI, case complexity index; ECCHO, Emergency Clinical County Hospital of Oradea; LOH, length of hospitalization

Section	No. (%) of beds 2021	National BUI	LOH 2020 standard	Case N	CCI 2020 standards	Maximum amount, 2021 lei (*euro)	Contracted amount, 2021 lei (*euro)	No. of cases contracted, 2021
A	B	C	D	E	F	G	H	I
Formula				[B]*[C]/[D]		[E]*[F]*WCC	[G]*P	[H]/[F] /WCC
General surgery	65 (24.43)	290.0	5.10	3696	2.6331	18043395.19 *3667058.61	14795584.06 *3006988.06	3031
Cardiovascular surgery	4 (1.5)	290.0	5.10	227	2.6331	1110362.78 *225665.14	910497.48 *185045.41	187
Thoracic surgery	6 (2.25)	290.0	5.10	341	2.6331	1665544.17 *338497.71	1365746.22 *277568.12	280
Vascular surgery	5 (1.87)	290.0	5.10	284	2.6331	1387953.48 *282081.43	1138121.85 *231306.77	233
Maxillofacial surgery	14 (5.26)	290.0	5.10	796	2.6331	3886269.73 *789828.00	3186741.18 *647658.96	653
Otorhinolaryngology	14 (5.26)	290.0	5.10	796	2.6331	3886269.73 *789828.00	3186741.18 *647658.96	653
Plastic surgery	20 (7.51)	290.0	5.10	1137	2.6331	5551813.91 *1128325.72	4552487.40 *925227.09	933
Burn unit	5 (1.87)	290.0	5.10	284	2.6331	1387953.48 *282081.43	1138121.85 *231306.77	233
Neurosurgery	25 (9.39)	290.0	5.10	1422	2.6331	6939767.38 *1410407.15	5690609.25 *1156533.86	1166
Ophthalmology	10 (3.75)	290.0	5.10	569	2.6331	2775906.95 *564162.86	2276243.70 *462613.54	466
Orthopaedics traumatology	63 (23.68)	290.0	5.10	3582	2.6331	17488213.80 *3554226.03	14340335.32 *2914465.35	2938
Urology	35 (13.15)	290.0	5.10	1990	2.6331	9715674.34 *1974570.02	7966852.95 *1619147.41	1632
Total surgical wards	266 (100)	290.0	5.10	15125	2.6331	73839124.95 *15006732.16	60548082.46 *12305520.37	12403

Note that, for the whole year 2021, the average 1 EUR value (expressed in lei) was 4.9204 lei. From Tables 6-7, it can be seen that the maximum amount contracted and the amount contracted by the hospital is higher when the LOH and CCI indicators achieved per ward are entered in the formula (SC=15837749.42 euro) compared to the indicators established at the national level (AC=15,006,732.16). This result is due to the fact that all the surgical wards included in the study recorded much better values than the national values. For the hospital, a lower LOH and a higher CCI than the national values lead to a higher contracted amount. The number of contracted cases is the result of the ratio of AC to WCC. The number of cases being directly influenced by the LOH; applying the LOH norms (5.10), the sections that exceed the LOH are advantaged, and the sections that have the LOH below the LOH norms are disadvantaged (Table 8).

**Table 8:** Difference between no. of cases contracted with LOH and CCI norms and no. of cases contracted with LOH and CCI realized hospital (ECCHO), 2021 *Difference between no. of cases contracted with LOH and CCI norms and no. of cases contracted with LOH and CCI hospital (ECCHO) realized

Section	LOH	No. (%) of cases contracted, 2021		No of cases/year*
		Standards	Made	
General surgery	6.15	3031 (100)	2513 (82.9)	517 cases
Cardiovascular surgery	11.15	187 (100)	85 (54.14)	101 cases
Thoracic surgery	6.78	280 (100)	210 (75)	69 cases
Vascular surgery	8.63	233 (100)	138 (59.22)	95 cases
Burn unit	10.79	653 (100)	862 (132)	123 cases
Neurosurgery	6.90	653 (100)	699 (107.04)	304 cases
Maxillofacial surgery	3.86	933 (100)	1091 (116.93)	-210 cases
Otorhinolaryngology	4.76	233 (100)	110 (47.21)	-47 cases
Plastic surgery	4.36	1166 (100)	862 (73.92)	-158 cases
Ophthalmology	1.58	466 (100)	1505 (322.96)	-1039 cases
Orthopaedics and traumatology	4.41	2938 (100)	3397 (115.62)	-460 cases
Urology	3.48	1632 (100)	2392 (146.56)	-760 cases
		12,403 (100)	13,865 (111.78)	-1467 cases

From the point of view of the amount contracted, taking into account the norm CCI (2.6331), it can be seen that the sections with CCI below the norm CCI are advantaged and those with CCI above the norm CCI are disadvantaged. Thus, if for a case with a norm CCI of 2.6331, the amount is 4.882 lei (WCC=1,854), for the same case with a realized CCI of 2.0639, the amount received per case will be 3.826 (Table 9).

**Table 9:** Difference between contracted amounts with CCI norms and contracted amounts with CCI hospital (ECCHO) realized (2021) * Difference between amounts contracted with CCI norms and amounts contracted with CCI hospital (ECCHO) realized

Section	CCI achieved	CCI standards	WCC	Amount/cash (realized CCI)	Difference/cases*
General surgery	2.0639	2.6331	1854	3826	1055
Maxillofacial surgery	2.3041	2.6331	1854	4272	610
Otorhinolaryngology	2.3293	2.6331	1854	4319	563
Ophthalmology	0.7873	2.6331	1854	1460	3422
Orthopaedics and traumatology	2.5419	2.6331	1854	4713	169
Cardiovascular surgery	4.4879	2.6331	1854	8321	-3439
Thoracic surgery	3.7496	2.6331	1854	6952	-2070
Vascular surgery	3.2096	2.6331	1854	5951	-1069
Plastic surgery	3.3724	2.6331	1854	6252	-1371
Burn unit	7.1219	2.6331	1854	13,204	-8322
Neurosurgery	3.8720	2.6331	1854	7179	-2297
Urology	2.6881	2.6331	1854	4984	-102

The study carried out was analyzed and exemplified for one year (2021) and how much the contracted amount is between the hospital and the health insurance company, if the indicators achieved by the hospital (DMS; ICM achieved) are entered in the calculation formula, compared to the established indicators by the rules of the national framework contract (DMS, ICM rules). Referring to the formula for calculating the contracted amount, the final amount will be higher if the DMS has a low value, and the ICM has a high value. Thus, in 2021, the hospital obtained a maximum contracted amount on the surgical wards analyzed of 831,017.26 EUR higher (by decreasing the DMS and increasing the ICM - achieved by the hospital) compared to the maximum contracted amount if the national values of these had been taken into account the indicators.

## Discussion

The present study focused on the quantitative and comparative analysis of hospital performance indicators used in the calculation of the amount contracted with the NHIS in order to quantify the results and evaluate the effectiveness of the measures taken in the surgical wards of the ECCHO compared to the annual average values at the national level, in the period 2012-2022. The analysis of qualitative and quantitative indicators of the efficiency of medical services in surgical wards reflects the efficiency of medical services and therefore of hospital management in the Romanian public health system. The results of our study are in line with the relevant findings of previous studies on the efficiency of the medical system [14,15,18-21]. Over the last 10 years, the hospital has undergone multiple transformations, from structural changes adapted to the needs of the population to rehabilitation and modernization of spaces (wards, laboratories, operating theatres), as well as high-performance equipment. All these changes have been made possible by efficient management and monthly monitoring of medical and financial activity, with the involvement of all heads of departments.

The activity of the surgical wards is dependent on the activity of the hospital's operating theatre, which is essential to ensure efficiency, patient safety, and quality of care [18,19]. An important activity carried out to improve hospital management is the generation of reports to monitor the organization, which form the basis for decision-making and resource allocation [3-15,19]. Hospital performance evaluation is based on the application of indicators and tools that summarize and present essential and timely information for managers [19]. By constantly monitoring and evaluating these indicators, hospitals can ensure that they provide the best possible care to their patients. In addition, this can lead to significant cost savings and improved efficiency, which can allow the hospital to treat more patients and improve the health of the community as a whole [19-21].

The LOH is one of the important operational indicators for hospital managers, used to assess hospital efficiency and productivity [21,22]. In various published studies, it is noted that this indicator is associated with a multitude of factors such as readmission rate, chance of repeated infections, burden of medical expenses for patient and family, and patient satisfaction [23,24]. Martinez et al. emphasized the importance of LOH alongside other indicators such as bed occupancy rate and number of patients in the emergency department [25]. In a study published in 2018 by Baek et al., a number of variables were identified that, when monitored continuously, lead to efficient use of hospital resources and decreased hospital length of stay [26]. Clinical protocols, on the other hand, lead to a reduction in complications with no effect on hospital length of stay and hospital costs. In our study, the average length of hospital stay on surgical wards in Romania in 2022 was 5.57 days, and in the hospital analyzed (ECCHO), it was 4.67 days. During the 11 years analyzed, the LOH decreased continuously, both nationally and in the ECCHO, with the exception of 2020 and 2021. During the two years of the COVID-19 pandemic, hospitals in Romania predominantly admitted only emergency cases and fewer scheduled cases, and due to the severity of the disease, these cases led to a slight increase in LOH. The severity of illness, complications of surgery, and low antibiotic use are factors leading to an increase in LOH, according to a study published by van Daalen et al. in 2017 [27].

Correct identification of these patients leads to better patient care but also to cost containment; the CCI measures the complexity and severity of cases that are treated in a given set of hospital care services [28]. The CCI is used in health insurance to determine the financing of health services in the contractual relationship between the health insurer and the hospital. The classification of patients into DRGs and the introduction of the DRG payment system allow linking the payment modality for hospitals to the efficient delivery of health services [29]. The MHI value achieved by the hospital has been on an upward trend during 2012-2022, being higher than the national MHI value, except for 2012 and 2013. If ICM and DMS were found in research published at the national level, they are not correlated with the amounts contracted with the insurance companies, restricting the possibility of a deep and thorough exploration of the findings resulting from our study.

The income obtained from the contract with the insurance company must ensure the operation of the hospital, with a significant share of the expenses being allocated to salaries, goods and services, medicines and sanitary materials, food, and current repairs. Without careful management of income and expenses, there is a risk that financial funds will be able to cover only basic expenses, at the expense of investments. Thus, at the level of each department, revenues must exceed expenses, the remaining amount being allocated to investments. Particular attention must be paid to the contractual relationship with the health insurance company for the provision of services, the contracted income being the hospital's main financial source.

Future research directions aim at expanding the analysis to all hospital departments and including more performance indicators in the study. Similar studies carried out by other hospitals would allow for inter-hospital comparisons, reflected in the performance of these hospitals.

Limits of the study: limiting the analysis performed only on surgical wards for acute conditions and including in the analysis only the performance indicators used in the formula for calculating the amount contracted with the insurance company; other relevant indicators being excluded (e.g., readmission to the hospital at less than 48-72 hours after discharge); carrying out the study only on a single clinical hospital, and other tertiary care, multi-station hospitals from other geographical regions of Romania were not analyzed.

## Conclusions

By including in the calculation formula the amount contracted by the hospital with the health insurance company (DRG system financing), the DMS, and ICM values, it is imperative to carefully monitor these indicators, both by the managerial and financial staff of the hospital and by department heads. The average length of hospitalization actually achieved in the wards with a surgical profile had a decreasing evolution in the 11 years analyzed, with a lower number of hospitalization days being recorded than at the national level (increased DMS: burns, cardiovascular surgery, thoracic surgery, and neurosurgery). The ICM had an increasing evolution throughout the analyzed period, the highest ICMs being on the wards with increased DMS. A lower DMS and a higher ICM achieved by the hospital compared to national averages lead to a higher contracted amount.

The improvement of hospital performance indicators is correlated with the increase in financing by the health insurance company. The annual increase in the contracted amount determines the contracting of an increased number of medical cases, which allows the hospital, from year to year, to increase its efficiency. Through the analysis, to offer a model of good practices for improving hospital financial performance, not only to decision-makers in Romanian hospitals but also to other countries where the contracting of health services between the hospital and the health insurance company follows these indicators. The study also shows how the values of the LOH and CCI indicators established in the legislation influence the number of cases contracted and the amount contracted. A more comprehensive analysis can provide complete information on the evolution of DRG contracting with the NHIS. The study carried out represents a useful analysis model for decision-makers in the hospital (managers, heads of departments), demonstrating the fact that the amount contracted with CAS can be increased by careful control of performance indicators.
